# Enhanced removal of As (V) from aqueous solution using modified hydrous ferric oxide nanoparticles

**DOI:** 10.1038/srep40765

**Published:** 2017-01-18

**Authors:** Lijuan Huo, Xibai Zeng, Shiming Su, Lingyu Bai, Yanan Wang

**Affiliations:** 1 Institute of Environment and Sustainable Development in Agriculture, Chinese Academy of Agricultural Sciences, Beijing, 100081, China; 2 College of Environment and Safety, Taiyuan University of Science and Technology, Taiyuan, Shanxi, 030024, China

## Abstract

Hydrous ferric oxide (HFO) is most effective with high treatment capacity on arsenate [As(V)] sorption although its transformation and aggregation nature need further improvement. Here, HFO nanoparticles with carboxymethyl cellulose (CMC) or starch as modifier was synthesized for the purpose of stability improvement and As(V) removal from water. Comparatively, CMC might be the optimum stabilizer for HFO nanoparticles because of more effective physical and chemical stability. The large-pore structure, high surface specific area, and the non-aggregated nature of CMC-HFO lead to increased adsorption sites, and thus high adsorption capacities of As(V) without pre-treatment (355 mg·g^−1^), which is much greater than those reported in previous studies. Second-order equation and dual-mode isotherm model could be successfully used to interpret the sorption kinetics and isotherms of As(V), respectively. FTIR, XPS and XRD analyses suggested that precipitation and surface complexation were primary mechanisms for As(V) removal by CMC modified HFO nanoparticles. A surface complexation model (SCM) was used to simulate As adsorption over pH 2.5–10.4. The predominant adsorbed arsenate species were modeled as bidentate binuclear surface complexes at low pH and as monodentate complexes at high pH. The immobilized arsenic remained stable when aging for 270 d at room temperature.

Arsenic (As) is a priority pollutant because of its highly toxicity and potential effects on public health and environmental safety worldwide. It has been detected widely in groundwater and soil and is particularly associated with waste from mining, petroleum refining, and ceramics manufacturing; agricultural chemicals; sewage sludge; and coal fly ash^1^. The efficiency of As cleanup by artificial adsorbents, such as iron (Fe) oxides (e.g., magnetite, ferrihydrite, goethite, and zero-valent iron [ZVI])^2^,^3^,^4^,^5^,^6^,^7^, activated alumina^8^,^9^, polymeric ligand exchangers^10^, red mud^11^, and activated carbon^12^,^13^, titanium oxide, has been extensively investigated in recent years. Fe(III) has high affinity for inorganic As species and selectively performs As sorption^1^,^12^. Inner sphere surface complexation can explain the strong interactions between As(V) and various Fe oxides^14^,^15^. Especially hydrous ferric oxide (HFO) is most effective for removing both As(III) and As(V) from aqueous solution because of its high specific surface area and iso-electric point^10^. However, the poorly crystalline HFO transforms into more crystalline iron forms, such as hematite or goethite, over time^16^, greatly diminishing its high reactivity with As and efficiency in its removal. Moreover, diffusion limitations within micrometer-sized HFO particles decrease their adsorption rate and available capacity^17^.

Recently, nanoadsorbents have made strong momentum in water and soil remediation engineering^18^,^19^,^20^. Nanoscale Fe oxide particles have larger specific surface area and potentially higher reactivity than bulk particles or natural minerals, which should confer greater sorption capacity. However, nanoparticles can undergo irreversible aggregation, which may reduce their sorption capacity and hinder their effectiveness^21^. Therefore, Fe oxides particles were decorated with various functional groups to enhance their dispersibility and performance^7^,^17^,^22^,^23^,^24^,^25^,^26^,^27^. Another unique advantage of well dispersed nanoparticles is that they can be directly delivered into contaminated soil or groundwater to facilitate *in situ* removal of target contaminants^28^. However, the modifier coatings for the HFO should play a maximum effectiveness for As(V) immobilization, while the negative ecological consequences also need to be considered.

Starch and carboxymethyl cellulose (CMC) are both low-cost and environmentally friendly, and they share similar macromolecular skeletons. Importantly, CMC carries carboxylate and hydroxyl groups^19^. They have been used as stabilizer materials in preparing ZVI, FeS, Fe-Mn oxides, or magnetite nanoparticles for heavy metals and organic contaminants^21^,^22^,^29^,^30^,^31^. It was reported the primary mechanism for binding CMC to Fe^2+^ was bidentate bridging while starch worked through steric stabilization^19^. Comparatively, Fe^3+^ is easier to complex with oxygen groups than Fe^2+ ^32. However, the understanding remains lacking on the binding mechanisms of Fe^3+^ and CMC or starch, as well as their performance in As(V) removal from aqueous solution.

In this work, in order to examine the effective removal of arsenate [As(V)] from aqueous solution, a series of HFO nanoparticles modified with various concentrations of starch or CMC were synthesized. The objectives were to (I) characterize the modified HFO nanoparticles and elucidate the stability mechanism; (II) test the effects of type and concentration of modifier, reaction time and solution pH on the effectiveness of As(V) sorption; (III) elucidate the As sorption kinetics, isotherm, and mechanism; (IV) test the effects of nanoparticle aging on As(V) immobilization and long-term stability; and (V) examine the reusability of the regenerated modified HFO nanoparticles for subsequent cycles of As(V) sorption.

## Results and Discussion

### Characterization of the modified HFO nanoparticles

**Figure 1:**
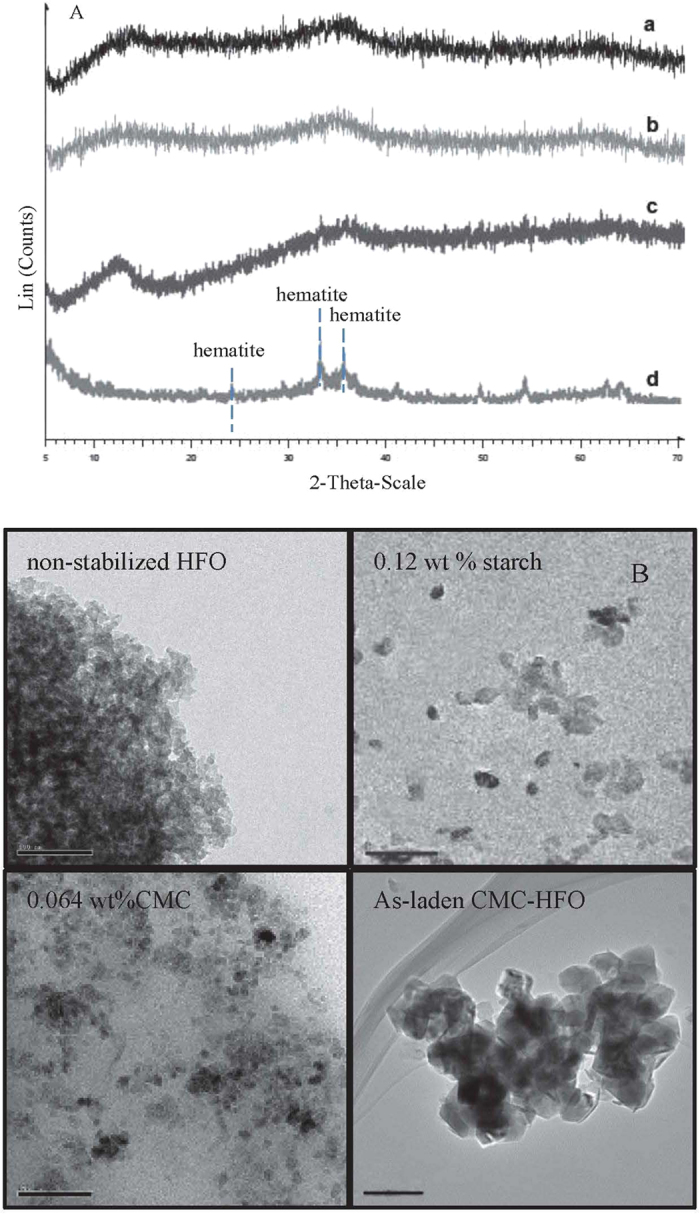
TEM image (**B**) and XRD spectra (**A**) of modified HFO nanoparticles: (a) 1 day and (b) 15days after HFO nanoparticles preparation with CMC stabilized (c) 1 day (d) 15 days after HFO particles preparation without modifier. The scale bar represents 100 nm.

The physical properties of the modified HFO particles, including their morphology, mean size, chemical and physical stability were measured by TEM, XRD, XPS, DLS and UV-vis analysis. The TEM micrographs of HFO particles prepared without a modifier, with 0.064 wt % CMC, and with 0.12 wt % starch were shown in Fig. 1. Bare HFO particles aggregated and precipitated quickly, appearing as large floccules (Figure S1 in Supplemental Information (SI)), while CMC or starch-modified HFO nanoparticles remained clearly discrete and well-dispersed in water (0.064 wt% CMC and 0.12 wt% starch). The mean size of the freshly prepared, CMC-stabilized nanoparticles was ~12 nm dispersed in CMC solution (Fig. 1B). DLS tests were also conducted on the modified HFO nanoparticles to obtain the dynamic“wet”particle size distribution. After 24 h of standing, the average hydrodynamic diameters of the bare HFO particles and those modified with 0.064% CMC and 0.12% starch were 1605, 216, and 283 nm, respectively. Figure S2 of SI displays the hydrodynamic size distributions of the particles at various CMC concentrations. At a CMC concentration of 0.064 wt%, fully stabilized HFO nanoparticles were obtained with a relatively narrow size distribution (>70% of the particles fall between 100 and 150 nm). At lower concentrations, the stabilizer serves conducts as a flocculating or bridging agent, to promote flocculation of particles flocculation or destabilization. When CMC or starch concentration exceeds a fixed value, the coating serves as a stabilizer, facilitating effective particle stabilization^30^. Yet, during a 180 days sediment test, the stabilized nanoparticles remained fully suspended. DLS revealed particle diameters of 227 and 378 nm for the CMC- and starch-modified particles, respectively. The measured ζ potential values of the HFO suspensions at pH 7.0 were 20.9, 1.3, and −38.9 mV for non-modified, starch-stabilized, and CMC-stabilized modified particles, respectively. Figure 1A also shows the XRD diffractograms of the prepared HFO nanoparticles in the presence and absence of stabilizer after prepared for 15 d. Hydrous Fe (III) oxides could convert to the crystalline Fe (III) oxides gradually^33^, but this did not happen for the CMC-modified nanoparticles. In the composites, no additional diffraction peaks appeared, suggesting that the particles are amorphous. However, the bare HFO nanoparticles contained a very small amount of crystalline hematite, which exhibit three weak peaks associated with the crystal structure of hematite at 24.06°, 33.14°, and 35.62° (Fig. 1d). UV-vis absorbance result (Figure S3) also illustrates the effects of CMC concentration on the HFO nanoparticles’ physical stability, and is described in S1 of SI. No significant changes in XRD spectra were found for the CMC–HFO nanoparticles, indicating that they are quite stable physically and chemically.

### Effects of modifier concentration on HFO nanoparticle stability and arsenic removal

**Figure 2:**
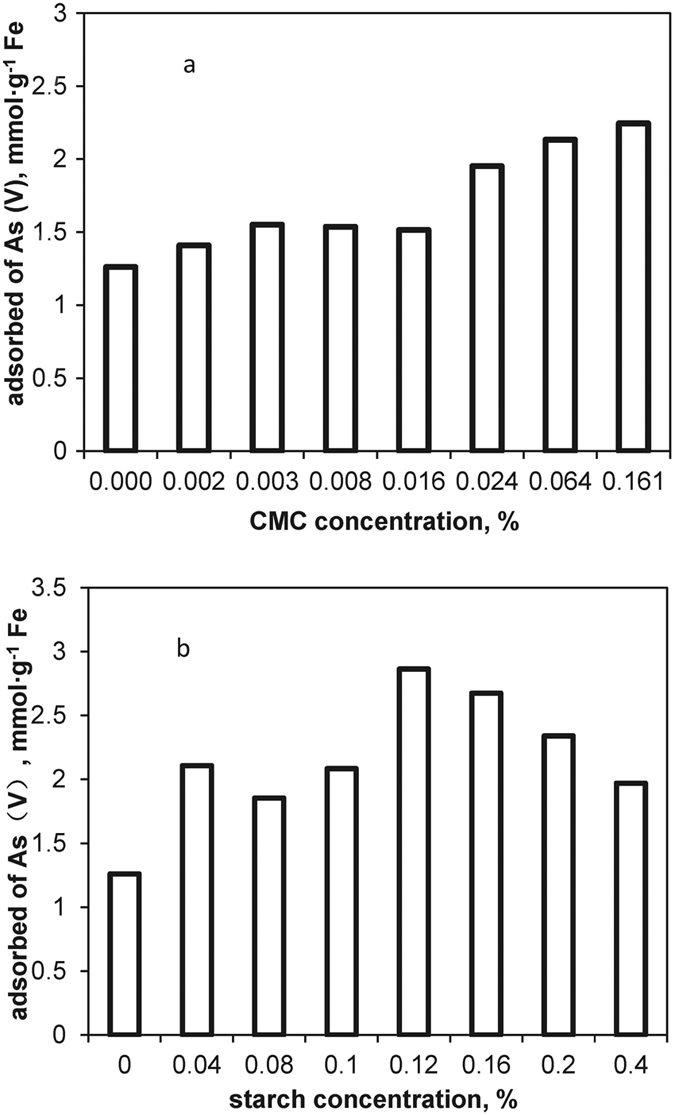
Effect of CMC (**a**) or starch (**b**) concentration on As (V) adsorption (q, mmol·g^−1^ Fe). Nanoparticle dosage = 100 mg·L^−1^ as Fe, initial As (V) concentration = 30 mg·L^−1^, equilibrium pH = 7.0, equilibrium time = 72 h.

Batch sorption tests were conducted to obtain the sorption capacity of As(V) on different modified HFO particles. Figure 2 shows the effect of stabilizer concentration on equilibrium uptake of As(V) by HFO nanoparticles. In general, As(V) uptake increased with elevated CMC and starch concentration in the ranges of 0–0.161 wt % and 0–0.4 wt %, respectively (Fig. 2). The modified composites gave much greater As(V) uptake than did bare HFO. The fully stabilized HFO nanoparticles (0.12 wt % starch and 0.064 wt % CMC) offered 2.31 and 1.69 times greater As(V) uptake than nonstabilized particles, respectively. This could be explained by the smaller size and the greater specific surface area of the modified nanoparticles. Larger CMC concentrations resulted in smaller size particles (Figure S2). Yean *et al*.^34^ and Auffan *et al*.^35^ reported that smaller-sized materials greatly improved As(V) adsorption capacity. As the particle size was reduced from 300 to 11 nm, the amount of adsorbed As(V) increased from 0.02 to 1.8 mmol·g^−1^ of magnetite (Fe_3_O_4_)^34^. Liang *et al*.^31^ observed that the sorption capacity was increased from 26 to 63 mg/g when the magnetite nanoparticle size ranged from ~200-75 nm. Nevertheless, As(V) uptake was inhibited when the starch concentration increased from 0.12 to 0.4 wt % (Fig. 2b). While the sorption capacity increased by 5% when CMC concentration increased from 0.064 to 0.161 wt % (Fig. 2a). Excessive uptake of CMC or starch molecules resulted in a denser coating on the nanoparticles’ surface, which was also used for zero iron, FeS and Fe-Pd nanoparticles preparation^21^,^29^,^30^. The associated elevated mass transfer resistance or sorption site blockage can inhibit As(V)’s access both kinetically and thermodynamically^30^.

Among the fully stabilized nanoparticles, those with 0.12 wt % starch offered 1.34 times greater As(V) removal than CMC stabilization, while CMC stabilization is associated with smaller particles. At the experimental pH of 7.0, H_2_AsO_4_^−^ and HAsO_4_^2−^ are the dominant arsenate species^36^. As CMC-modified HFO nanoparticles have a highly negatively charged surface (ζ potential −38.9 mV), the anions would need to overcome the energy barrier created by electrostatic repulsion in order to undergo sorption. Although starches can stabilize HFO nanoparticles in aqueous solutions, their effectiveness is limited by weaker, interfacial bonding with the particle surface and their lower-magnitude ζ potentials than CMC. In contrast, CMC consists of oxygen-containing functional groups (e.g., –COOH, –OH, and –CO) and stabilizing, hydrocarbon chains, which can interact with particles much more strongly than starch. CMC thus serves as a more effective capping agent^37^. The anchoring groups can attach chemically or physically onto the HFO nanoparticle surface, while the stabilizing chains can freely rotate and take on varied configurations in water^38^. He *et al*.^39^ concluded that CMC molecules complex with Fe^2+^ to give the precursor CMC- Fe^2+^ in the synthesis of nanoparticles. The complexation did not appear to hinder significantly the subsequent reduction or co-precipitation of the Fe^2+^ possibly owing to the rather bulky and loose structure of the CMC molecules. Thus, the primary role of CMC lies in facilitating the subsequent nucleation and growth of the composites, thereby prevent the aggregation of nanoparticles via electrosteric stabilization^19^. Moreover, as described above, CMC requires a lower concentration than starch to achieve full stabilization of HFO nanoparticles (0.064 and 0.16 wt % for CMC and starch, respectively). When all factors, including As(V) sorption, physical stability, and environmental effects, are analyzed together, it could be easily inferred that CMC might be the optimum stabilizer for HFO nanoparticles. The optimal concentration of CMC was determined to be 0.064 wt %.

### Arsenic sorption kinetics and isotherm

**Figure 3:**
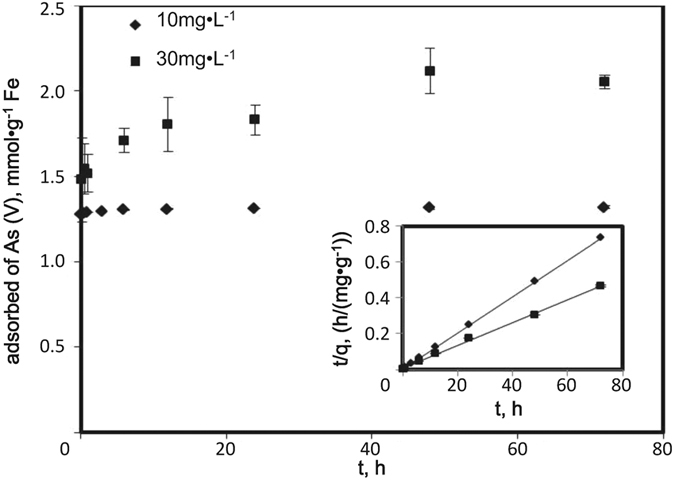
Uptake of As(V) by CMC-HFO nanoparticles. Initial As(V) = 10 and 30 mg·L^−1^, HFO particles as Fe = 100 mg·L^−1^, CMC = 0.064% (w/w), initial pH (pH_0_) = 6.5, and final pH (pH_f_) = 7.0. Control tests were performed with no HFO (0.064 wt% CMC). Inset is the plot of t/q_t_ versus t using linear regression fitting a pseudo-second-order model.

The effect of As(V) sorption kinetics and isotherm on CMC modified HFO nanoparticles were evaluated. Figure 3 shows the results of kinetics tests on CMC-modified HFO nanoparticles with Fe at 100 mg·L^−1^ and As(V) at an initial concentration of 10 and 30 mg·L^−1^. Overall, the sorption rate is characterized with a rapid initial rate (at <1 h) followed by a rather slower phase until reaching equilibrium at 72 h. At equilibrium, 99% of the As(V) had been removed from the aqueous when the initial As(V) concentration was 10 mg·L^−1^. The first-order, second-order and intraparticle diffusion models were used to simulate the kinetics data; Fig. 3 also provides best fitting curves to these models. The second-order kinetics was able to adequately interpret the batch kinetic data (R^2^ = 0.997 and 1). It was consistent with the findings by Zhu *et al*.^40^ and Goh *et al*.^41^, who modeled As(V) sorption kinetics by hydrous ferric oxide and Mg/Al layered double hydroxide. The results indicated that the rate-limiting step is sorption rather than diffusion.

**Figure 4:**
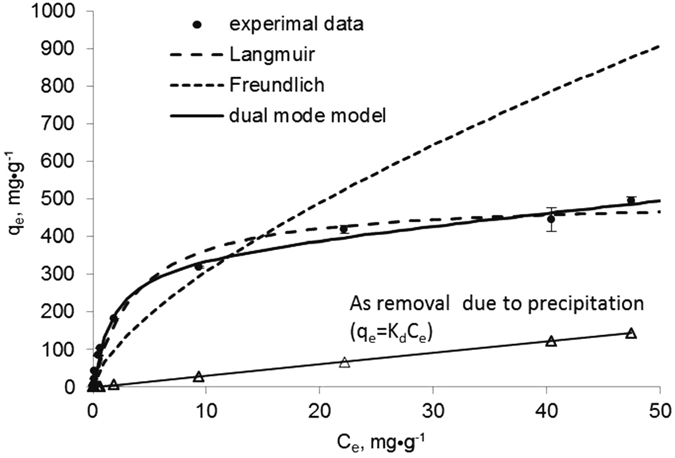
Arsenic sorption isotherm of CMC- HFO nanoparticles. HFO particles as Fe = 100 mg·L^−1^, initial As(V) concentration = 0.1–100 mg·L^−1^, initial pH = 7.0. Symbols: experimental data; lines: model fittings.

Figure 4 shows the As(V) sorption isotherm for the modified HFO nanoparticles at pH 7.0. The experimental data were fit to the classical Langmuir and Freundlich models. The Langmuir model outperformed the Freundlich model (R^2^ = 0.9873 and 0.9086, respectively), consistent with findings by An *et al*.^42^. For mechanistic soundness, a dual-mode isotherm model that incorporates both precipitation and adsorption (i.e., ion exchange and surface complexation) was applied to simulate the experimental data^30^:


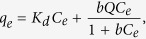


where *q*_*e*_, *C*_*e*_, *K*_*d*_, *b*, and *Q* represent total As(V) uptake (mg·g^−1^), equilibrium concentration of As(V) in solution (mg·L^−1^), the linear distribution coefficient (L·mg^−1^) associated with precipitation, the Langmuir affinity constant (L·mg^−1^), and the Langmuir maximum sorption capacity (mg·g^−1^), respectively. In theory, the first and second terms represent As(V) precipitation (which is considered to be linearly related to *C*_*e*_) and adsorption, respectively. The nonlinear fitting verifies that the dual-mode model best parameterizes the experimental data, providing the best fit (R^2^ = 0.9966) and the adsorption capacity 355.3 mg·g^−1^ as Fe. The same formula has been successfully used to model sorption of mercury onto FeS nanoparticles^30^. Figure 4 also illustrates precipitation becomes more important following the increased As concentrations. At lower concentration of As(V) (Ce < 9.39 mg·L^−1^, initial As/Fe < 0.4), more than 97% of the observed As(V) removal was attributed to adsorption. Increased As(V) loading saturated the HFO nanoparticles’ adsorption capacity, giving rise to precipitation: at Ce > 40 mg·L^−1^ (initial As/Fe > 0.6), 15%–28% of As(V) removal was due to precipitation. Similar findings were reported by Tokoro *et al*.^6^, who studied As(V) coprecipitation with HFO at pH 5 and 7. They concluded that at As/Fe < 0.4, adsorption mainly caused As(V) removal, whereas at As/Fe > 0.4, ferric arsenate formed a surface complex.

Table 1 compares the Q-values of the present systems’ by some reported procedures. Decorating HFO with CMC performs significantly improved adsorption capacity for As(V) compared with those of previously reported. The maximum adsorption capacity enhanced at least 1.43 times than the values reported in previous studies as we know. Furthermore, this feature of CMC modified HFO nanoparticle developed in this work was synthesized through direct synthesis at normal experiments, which is much more convenient and cost-effective for large scale operations. Taking As(V) sorption capacity and the preparation costs into account, the CMC-HFO nanoparticle is a rather promising alternative for As(V) removal. However, the CMC-to-HFO ratio will need to be adjusted such that the resulting CMC-HFO nanoparticles, referred to as flocculated nanoparticles, can have the advantage of the high specific surface area and easy separation by gravity-settling.

### Arsenic sorption mechanism of CMC modified nanoparticles

**Figure 5:**
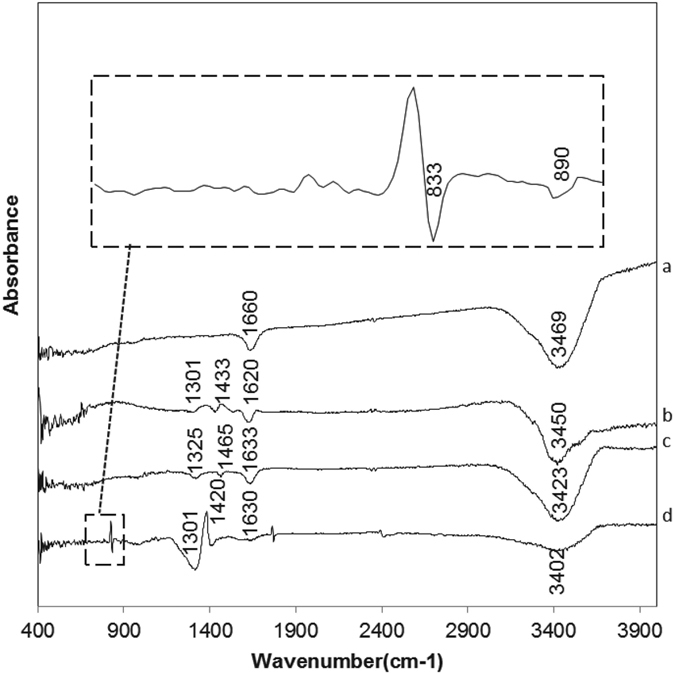
FT-IR spectra of (a) HFO (b) neat CMC (c) CMC-HFO nanoparticles (d) As laden CMC-HFO nanoparticles.

FTIR spectroscopy was used to elucidate the mixtures’ stabilization mechanisms and clarify the properties of the chemical bonding between HFO, CMC, and As(V). Figure 5 presents the FTIR spectra of neat CMC, bare HFO, and modified HFO before and after As(V) sorption. The spectrum for bare HFO particles showed a peak at 1660 cm^−1^ and another at 3469 cm^−1^ (Fig. 5a), corresponding to the O–H bond from H_2_O^48^. It is reported that the pK_a_ value of CMC is 4.3 4, and in all synthesis procedures, the pH was above 5.3. Therefore, the carboxylic acid groups and hydroxyl of CMC are expected to be almost fully disassociated. The carboxylic acid and hydroxyl groups interact strongly with the Fe^3+^ cations. Three FTIR peaks were observed for CMC–HFO nanoparticles at wavenumbers 1465, 1633 and 3423 cm^−1^ because of interactions between COO^−^ (1465 and 1633 cm^−1^) or –OH (3423 cm^−1^) and Fe (Fig. 5c). The stretching frequencies for the functional groups of CMC are expected to shift significantly if CMC molecules are adsorbed to the surface of the Fe nanoparticles^49^; thus, it is worth noting that the –OH stretching band shifts from 3450 cm^−1^ to 3423 cm^−1^ for CMC and CMC–HFO particles, respectively. This observation demonstrates that an enhanced intermolecular hydrogen bond is formed between CMC and the Fe^3+ ^19. The peaks at 1620 and 1433 cm^−1^ for CMC are assigned to asymmetric and symmetric COO^−^ groups, respectively; these peaks were shifted to 1633 and 1325 cm^−1^, respectively, for CMC–HFO (Fig. 5c). The difference between the asymmetric and symmetric stretches [Δ*v* = Δ(asym) − Δ(sym)] of the carboxylate group was 308 cm^−1 ^19,30, suggesting that CMC–Fe^3+^ binding is governed by monodentate bridging. In addition, compared with that of CMC, the bond strength of the –OH group increased for CMC–HFO. This increase indicated the existence of an enhanced intermolecular hydrogen bond between CMC and Fe. An *et al*.^42^ reported that the peaks associated with the H-bonded –OH groups of CMC were greatly intensified for CMC-bearing magnetite nanoparticles, which was consistent with our FTIR results.

Comparing the spectrum of the CMC-HFO before and after As(V) sorption, new broad bands were evident at ~833 and ~890 cm^−1^ for the As-laden HFO nanoparticles (Fig. 5d). The band at ~833 cm^−1^ was due to coordination of the stretching As–O vibration with the Fe atom, i.e., As–O–Fe. Jia *et al*.^50^ conducted an FTIR study on sorption of As(V) to ferrihydrite and reported that poorly crystalline ferric arsenate shows a strong, well-resolved band at ~838 cm^−1^, which was assigned to Fe–O–As. They concluded that within the crystalline ferric arsenate structure, AsO_4_ tetrahedra and FeO_4_(OH_2_)_2_ octahedra connected at alternate vertices; the band at ~890 cm^−1^ (Fig. 5d) was assigned to uncomplexed/unprotonated As–O. For arsenate sorbed on amorphous Fe oxide, Goldberg and Johnston^51^ reported the existence of two distinct bands corresponding to surface-complexed and non-surface-complexed As–O groups. Moreover, the FTIR spectra showed similar absorption band characteristics to those found in the present study, such as those for the –OH group as well as the asymmetric and symmetric stretches of COO^−^ groups (3402, 1630, and 1301 cm^−1^, respectively). However, the –OH stretching band shifted from 3423 cm^−1^ to 3402 cm^−1^ for CMC-HFO nanoparticles and As-laden CMC-HFO, respectively, while the frequency of the interaction between –OH and Fe commensurately decreased. The results showed a weakened Fe–OH peak when As (V) was adsorbed onto the CMC–HFO nanoparticles; yet, the frequency of the symmetric stretch of the COO^−^ group increased from 1325 to 1301 cm^−1^ for bare and As-laden CMC-HFO, respectively. These changes were attributed to the complexation of As onto the COO^−^ group of CMC because the abundance of –OH and COO^−^ groups in CMC increased the number of available adsorption sites for As(V).

Figure S4 in SI presents XRD spectra of CMC–HFO particles mixed with As(V) and aged 30 d and 270 d; they show a broad band at ~34° 2θ. After 30 d of aging, a peak also appeared at 2θ values of ~13°, corresponding to the characteristic peak of symplesite, but no other peaks were found in the 30-day spectrum. After 270 d of aging at an initial pH of 7.0 at 25 °C, a weak band emerges at ~28° 2θ, indicating the development of the ferric arsenate phase. The intensity of the band at ~13° was enhanced after 270 d of aging. Jia *et al*.^52^ indicated that poorly crystalline ferric arsenate shows two broad XRD bands located at ~28° and ~58° 2θ when the system was equilibrated for 2 weeks at 75 °C and pH 3.0.

**Figure 6:**
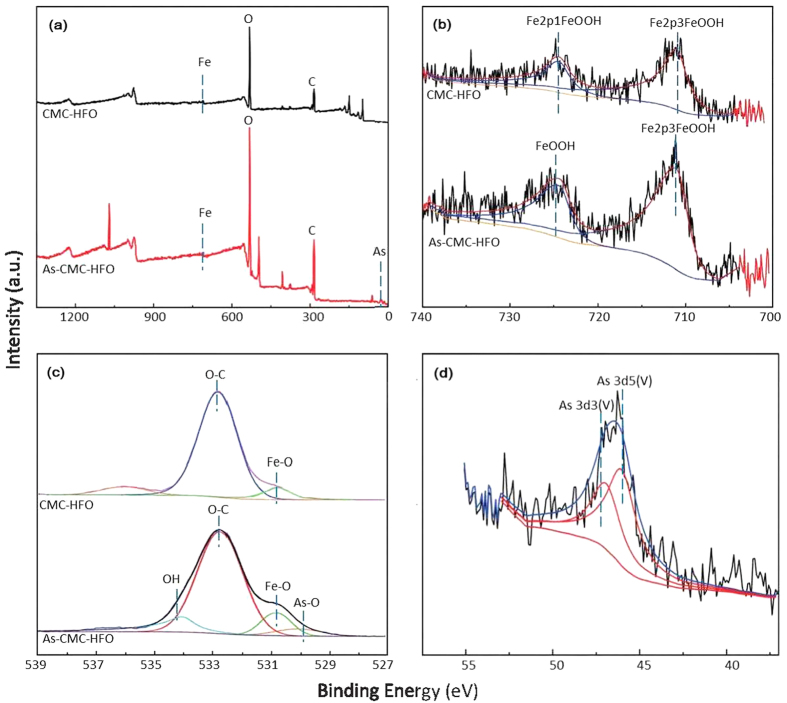
XPS survey scan (**a**) and corresponding high resolution spectra of Fe 2p (**b**) O1s (**c**) and As 3d (**d**) for fresh and As(V)-loaded CMC-HFO.

XPS was used to confirm the surface structure for fresh and As(V)-loaded CMC-HFO composites. The wide scan XPS spectrum of the CMC-HFO composites (Fig. 6a) shows the photoelectron lines at binding energies of about 284.0, 530.0, 710.0, and 46.2 eV are attributed to C1s, O1s, Fe2p, and As3d respectively. Two photoelectron peaks located at 711.3 and 724.5 eV are found in the Fe 2p spectrum (Fig. 6b), which can be assigned to the Fe2p_3/2_ and Fe 2p_1/2_ of FeOOH, respectively. The O1s spectrum (Fig. 6c) can be deconvoluted into two peaks at 530.7and 532.7, which are attributed to the binding energies of Fe–O, and C–O^41^. The high resolution XPS As 3d spectrum after As(V) adsorption showed a remarkable increase in peak size located at 46.2 eV (Fig. 6d), and signified the successful As(V) binding to CMC-HFO nanoparticles. Moreover, the O 1 s spectrum (Fig. 6c) is deconvoluted into four components of Fe–O, As–O, C–O and –OH. The appearance of As–O peaks at 529.9 eV indicates the adsorption of arsenic onto the surface of the adsorbents. A significant peak at 534.3 eV appears loaded with As(V) on CMC-HFO composites^43^, implying the alteration of oxygen constituents of CMC-HFO after As(V) sorption (Fig. 6c). A significant increase of the Fe2p spectra intensity of CMC-HFO and a spectra shift (711.3 eV) were observed following As(V) sorption, showing strong interactions between As(V) and Fe atoms. Thus Fe atoms likely played an important role in As(V) sorption.

TEM spectra of As-laden CMC-HFO nanoparticles after reaction (Fig. 1) showed that the agglomeration as well as size of the nanoparticles increased probably due to the formation of the adsorption/co-precipitation of As(V) on CMC-HFO particles surface^53^. This result is consistent with the results of XRD, XPS, FTIR and dual-mode isotherm model.

### Effects of pH on arsenic removal

**Figure 7:**
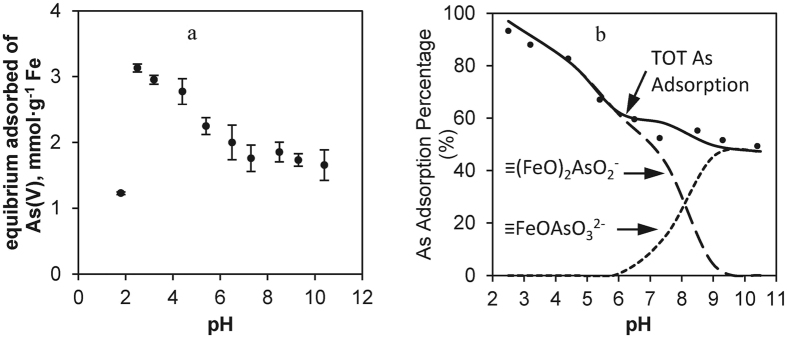
(**a**) Arsenic removal as a function of pH. (**b**) speciation of adsorbed arsenate at total As = 10 mg·L^−1^ using SCM model. HFO nanoparticles = 100 mg·L^−1^ as Fe. Data are shown as symbols and model simulations are shown as lines.

The formation of HFO particles via surface complexation and surface precipitation are pH-dependent processes. Solution pH can affect both arsenate speciation and surface charge of CMC–HFO nanoparticles and further change the sorption capacity As(V). Figure 7 shows the effects of equilibrium pH on As(V) removal by CMC–HFO. As(V) uptake decreased from 3.1 to 1.9 mmol·g^−1^ Fe as pH increased from 2.5 to 6.5 and then remained at ~1.8 mmol·g^−1^ Fe at pH 6.5–10.4. Increasing pH has been reported to decrease As(V) adsorption on ferric hydroxide^16^,^32^,^41^,^54^. The surface charge of nanoparticles remained negative at pH values as low as 2.5 (ζ potential −0.65 mV) as shown in Figure S5. It could be inferred that the pH_PZC_ of stabilized HFO nanoparticles in our study was <2.5 and that at pH > pHpzc, the HFO surface was negatively charged. The higher the solution pH, the more negatively charged the surface of the HFO nanoparticles is, and the less sorption of As(V) onto the HFO nanoparticles occurs. As shown in Figure S6, at pH < 6.5, the HFO particles increasingly dissolved, and the concentration of Fe ions, which react with As (V) in solution, increased (Figure S6). In addition, the concentrations of As species are pH-dependent: the acid dissociation constants (pK_a1_, pK_a2_, and pK_a3_) for arsenate are 2.2, 6.9, and 12, respectively^10^. As a result, an excess of OH^−^ accumulates at the HFO particle–solution interface, promoting the conversion of H_2_AsO_4_^−^ from the bulk solution to HAsO_4_^2−^ at the HFO nanoparticle surface. At pH > 7.3, although the more adsorbable HAsO_4_^2−^ ions predominate, the competition from OH^−^ ions becomes increasingly fierce, resulting in reduced As uptake with increasing pH.

However, when pH decreased to 1.8, adsorbed As(V) sharply decreased to 1.2 mmol·g^−1^ Fe. It was attributed to the dominant As species H_3_AsO_4_ at pH < 2.2, which hindered the adsorption of As(V) on the particles^54^. It can be concluded that an effective removal of monovalent As(V) can be accompanied by using CMC-HFO nanoparticles, which is consistent with the reports from Ghimire *et al*.^54^. The optimum adsorption conditions for arsenate are pH = 2.5 and the maximum removal percentage was around 93.3%.

A surface complexation model (SCM) with the double-layer model (2-pk DLM) was used to describe the arsenic sorption edges. Similar surface complexes have been used in previous studies^16^,^55^,^56^. The surface sites (≡FeOH) were considered similar to those of two-line ferrihydrite^55^. Constants for protonation of the surface hydroxyl groups and aqueous species were taken from previous studies shown in Table 2. To simplify the model, only nonprotonated bidentate surface complexes were considered^55^. The intrinsic As(V) surface complexation constants for As(V) adsorption optimized with DLM are shown in Table 2 using Minteq software.

The SCM fits the experimental data for As(V) adsorption percent over the range of pH 2.5–10.4 (Fig. 7b). Better agreement between the model and the experimental data are obtained in a pH gradient. The percentage distributions of arsenate species are ~50.0% for H_2_AsO_4_^−^ and ~9.0% for HAsO_4_^2−^ in As3d spectra at pH 7.0. The peaks corresponding to these species are centered at 47.2 and 45.5 eV respectively in XPS spectra^53^. The SCM model results suggested that the intrinsic binuclear, bidentate surface complex ≡(FeO)_2_AsO_2_^−^ was predicted to be the dominant form of adsorbed arsenate over pH 2.5–5.9 and the monodentate complex ≡FeOAsO_3_^2−^ was predicted to dominate above pH 6.8 (Fig. 7b). The results were consistent with those previously reported. Zeng *et al*.^55^ concluded that the predominant, adsorbed arsenate and phosphate species on iron oxide-based sorbent were modeled as bidentate, binuclear surface complexes at low pH and as monodentate complexes at high pH.

After 270 d of aging at room temperature, sorption capacity changed from 3.13 to 2.08, from 1.99 to 1.63, and from 1.66 to 1.70 mmol/g for pH 2.5, 7.1, and 11.0, respectively. Only small changes in sorption capacity were associated with the pH changes. The pH values revert towards neutrality after aging: the pH values of the solutions that start at pH 2.5 and 11 change to 3.3 and 10.2, respectively, after 270 d.

### Regeneration

The HFO nanoparticles after the adsorption test can be regenerated by stirring with 0.1 mol·L^−1^ NaOH solution at 25 °C for 4 h respectively^26^,^41^,^43^. Meanwhile the recyclability of CMC-HFO for As(V) adsorption was investigated by repeating the adsorption/desorption process three times, the results of which are presented in Figure S7. The adsorption efficiency of CMC-HFO for As(V) showed no significant loss after three successive cycles (99.7–94.9%). The reusability of CMC-HFO demonstrates its advantage for As(V) removal from drinking water. The deterioration in capacity for the second and third cycle is 2.0% and 5% for As(V), respectively. These results showed that CMC-HFO has sufficient chemical stability over several sorption-desorption repetitions.

To further confirm the effectiveness of HFO nanoparticles in arsenic removal from bodies of water, the adsorption experiments were performed in wastewater samples from Realgar mine tailings with an initial arsenic concentration of 38.2 mg·L^−1^, since the composition of real wastewater is more complex. Although large amounts of other species exist in the wastewater (detailed information is shown in Table S1, SI), modified HFO nanoparticles still show an excellent performance with an adsorption efficiency of ~90.5% for As(V) (Figure S8). For continuous treatment by CMC-HFO for three times, the concentration can be decreased to 5.6 μg·L^−1^, far below the standard of 10 μg·L^−1^ suggested by WHO. These results further confirm applicability of CMC-HFO nanoparticles in arsenic removal from wastewater without pre-treatment.

## Conclusions

This study addressed the potential for and viability issues of the nanoparticles for As(V) removal from water. The particle stabilization technique not only facilitated deliverability, but increased the As(V) sorption capacity by up to 1.69 times with 0.064 wt% CMC. However, excessive CMC can inhibit As(V)’s sorbed to particle surface sites both kinetically and thermodynamically. The rate-limiting step of As(V) removal rates for CMC–HFO nanoparticles is sorption rather than diffusion. At initial As/Fe < 0.4, more than 97% removal was attributed to adsorption, but as As(V) loading increased, the adsorption capacity of the HFO nanoparticles became saturated, leading to chemical precipitation: at initial As/Fe > 0.6, precipitation caused 15–28% of As(V) removal. Furthermore, the FTIR, XRD and XPS analysis also supports that CMC–HFO takes up As(V) through concurrent precipitation (formation of crystalline ferric arsenate) and adsorption. Media pH was an important factor controlling the arsenate species present at the CMC–HFO nanoparticle surface. This study also demonstrated that the particles can be applied in a device to continuously treat arsenic-polluted water from a realgar mine, lowering concentrations from 38.2 mg·L^−1^ to 5.6 μg·L^−1^ below the WHO standard. The CMC- modified HFO nanoparticles may designed to be deliverable into contaminated soil or sediment to facilitate *in situ* immobilization of As(V).

## Methods

### Materials

Hydrous ferric oxide (HFO) nanoparticles were modified by CMC or starch via a water-based approach^57^. The modification was conducted in a 1 L flask in the presence of 1 wt % starch or CMC stock solution, which was prepared at room temperature following the method from He *et al*.^39^. The detailed method is described in S2 in SI. The final suspension of HFO nanoparticles was dialyzed as rapidly as possible to remove electrolytes until an electric conductivity (EC) value was below 10 μs/cm.

### Physical characterization of modified HFO nanoparticles

The modified HFO nanoparticles were characterized using transmission electron microscopy (TEM, JEM-2010 JEOL Ltd., Tokyo, Japan), X-ray diffraction (XRD, Bruker AXS, Inc., Madison, WI, USA), X-ray photoelectron spectroscopy (XPS, Thermo escalab 250Xi, USA) and Fourier transform infrared (FTIR) spectroscopy. Dynamic light scattering (DLS) analysis the dynamic particle size distribution and ζ potential of HFO particles employed a Malvern Zetasizer Nano ZS (Malvern Instruments, Worcestershire, UK). Section S3 of SI provides details of the methods.

### Effects of the modifier type and concentration on HFO nanoparticle stability and As sorption

To study the modifier’s effects, HFO particles were prepared at a fixed HFO concentration of 100 mg·L^−1^ as Fe and modified with different concentrations of CMC or starch (0–0.161 wt % and 0–0.4 wt%, respectively). To qualify the nanoparticles’ physical stability/settleability after 2 d aging, the supernatant samples’ visual transparency was then compared using UV-vis absorbance (UV-2550PC, Shimadzu Corporation, Japan). Meanwhile, the supernatant samples were digested with 12 M HCl for 5 min, which completely dissolved the nanoparticles, and then analyzed for total Fe.

### Arsenic sorption tests

Batch kinetics experiments were conducted in 50 mL centrifuge tubes. As(V) stock solution was prepared by dissolving sodium arsenate dodecahydrate (Na_3_AsO_4_ · 12H_2_O) into Milli-Q water. Each reactor contained 8 mL of CMC-stabilized or non-stabilized HFO suspension (500 mg·L^−1^), which was then diluted by adding 32 mL of a solution, yielding a final solution containing 100 mg·L^−1^ of HFO. Sorption of As(V) was initiated by injecting an stock solution into the reactors, resulting in an initial As(V) concentration of 10 and 30 mg·L^−1^. The pH of the mixture was kept to 7.0 with 0.1 M HNO_3_ and 0.1 M NaOH. The mixtures were continuously shaken on a thermostatic shaker at 170 rpm and at 25 ± 1 °C. At predetermined times, duplicate vials were sacrificially sampled. The samples were then filtered through a 25 nm membrane filters (VSWP, Millipore, USA) and then analyzed for arsenic concentration in the filtrates. The filtration was able to retain >96% of the nanoparticles as determined by analyzing total iron in the filtrate, without retaining any soluble As. The final pH, measured at the end of the experiments, is reported. The same test procedure of equilibrium sorption test was followed except the varying initial As(V) concentration 0.1–100 mg·L^−1^. To study the pH effect, the sorption tests were carried out at pH from 1.8–10.4. Control sorption experiments with DI water, CMC or starch but without particles were performed. The results showed that there was nearly no loss of arsenate in solution during the 72 h tests at pH in the study (Figure S9 of SI). Experiments were repeated three times, and all of the data are the average of the three.

### Analytical methods

Arsenic concentration in the solution was analyzed using a hydride generation atomic fluorescence spectrometer (HG-AFS9120, Titan Instruments, Beijing, China). The detection limits for As were 0.02–0.04 mg·L^−1^. Iron concentration in solution was analyzed using a inductively coupled plasma mass spectrometer (ICP, Optima 5300DV, USA)

## Additional Information

**How to cite this article**: Huo, L. *et al*. Enhanced removal of As (V) from aqueous solution using modified hydrous ferric oxide nanoparticles. *Sci. Rep.* **7**, 40765; doi: 10.1038/srep40765 (2017).

**Publisher's note:** Springer Nature remains neutral with regard to jurisdictional claims in published maps and institutional affiliations.

## Supplementary Material

Supplemental information

## Figures and Tables

**Table 1:** Comparison of As(V) adsorption capacity.

Sorbent	As(V) Q_max_ (mg/g)	Ref.
HFO coated activated carbon (7.5% Fe)	260	(Jang)^1^
ultrafine α-Fe_2_O_3_ nanoparticles	47	(Tang)^4^
γ-alumina nanocrystalline (0.25 g)	27.75	(Li)^8^
polymeric ligand exchanger (0.15 g)	92	(An)^10^
amorphous iron oxide	157.33	(Dixit)^16^
zero iron encapsulated chitosan nanospheres(0.5 g·L^−1^)	119	(Gupta)^17^
ionically modified magnetic nanomaterials (120 mg)	50.5	(Badruddoza)^26^
magnetite nanoparticles with starch (0.1 g·L^−1^ Fe)	62	(Liang)^31^
HFO Flocs	139	(Raven)^33^
magnetite nanoparticles with CMC (0.57 g·L^−1^ Fe)	248	(An)^42^
magnetite nanocrystals (0.1 g·L^−1^ Fe)	241	(Yean)^34^
γ-Fe_2_O_3_ Nanoparticles @ macroporous silica	248	(Yang)^43^
Mg/Al Layered Double Hydroxide (0.4 g·L^−1^)	85	(Goh)^41^
functionalized graphene sheets based electrodes	142	(Mishra)^44^
β-FeOOH@GO-COOH composite (1 mg)	45.7	(Chen)^45^
iron oxide-graphene nanocomposite (5 mg)	172.1	(Mishra)^46^
Fe(III) loaded chelating resins (0.2 g)	55.5	(Matsunaga)^47^
HFO-CMC nanoparticle (0.1 g·L^−1^ Fe)	355	This work

**Table 2:** Material properties, experimental conditions, reactions and parameters used in surface complexation modeling for adsorption of arsenate on HFO nanoparticles.

material properties and experimental Conditions	
specific surface area (m^2^·g^−1^)	600^a^
solid concentration (g·L^−1^)	0.1
equilibration time (h)	72
site concentration (mM)	1.5
	1.1
sorption density (mol As per mol Fe)	0.26^b^
intrinsic surface complexation constants	
reaction	Log K
surface acidity reaction	
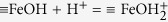	−6.51^c^
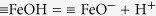	−8.93^c^
Arsenate adsorption constants	
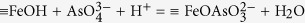	18.9^d^
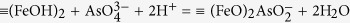	27.1^d^

^a^Assumed value^56^;

^b^Obtained from sorption isotherm;

^c^Reference^16^;

^d^This study.
