# Supplementary material for: Enhanced removal of As (V) from aqueous solution using modified hydrous ferric oxide nanoparticles
Source: Sci Rep. 2017 Jan 18;7:40765. doi: 10.1038/srep40765 (PMC5241682; doi:10.1038/srep40765)
Supplement: Supplemental information [file srep40765-s1.doc]

**Enhanced removal of As (V) from aqueous solution using modified hydrous ferric oxide nanoparticles**

Lijuan Huo^1,2^, Xibai Zeng^1 *^, Shiming Su^1^, Lingyu Bai^1^, Yanan Wang^1^

1. Institute of Environment and Sustainable Development in Agriculture, Chinese Academy of Agricultural Sciences, Beijing, 100081, China

2. College of Environment and Safety, Taiyuan University of Science and Technology, Taiyuan, Shanxi, 030024, China

**Appendix A. Supplemental Information** for Publication in

The Journal of Scientific Reports

^*^ Corresponding author. E-mail: zengxibai@caas.cn; Phone: 086-10-82105612

**Supplemental material:**

**Table: 1**

**Figure: 9**

**S1 Characterization of the modified HFO nanoparticles**

Figure s1 demonstrates the physical stability and settleability of HFO nanoparticles prepared at a particle concentration of 100 mg·L^-1^ as Fe but with various concentration of CMC ranging from 0 to 0.161 wt %. Based on the results, this study focused on fully stabilized HFO nanoparticle suspensions, which were prepared at a final concentration of 100 mg·L^-1^ as Fe and 0.064 wt % CMC (or 0.12 wt% starch). Thus, as described in Figure s1, 0.064 wt% CMC can be regarded as the critical stabilization concentration for 100 mg·L^-1^ as Fe HFO nanoparticles. At a CMC concentration lower 0.064 wt %, rapid flocculation and settling of the particles were observed.

Figure s3 illustrates the effects of CMC concentration on the HFO nanoparticles’ physical stability via UV-vis absorbance. For the bare HFO particles, no absorbance was observed at wavelengths 190–280 nm, and the solutions appeared transparent. After 15 days of gravitational setting, 92% of the bare HFO particles had sedimented, compared with 1.9% and 2.5% sedimentation at CMC concentrations of 0.064 and 0.161 wt %, respectively. The results indicated that HFO nanoparticle suspensions prepared at final concentrations of 100 mg·L^-1^ as Fe and 0.064 wt % CMC would be fully stabilized. HFO crossed linked with adsorption of starch or CMC macromolecules to nanoparticles can greatly influence their surface characteristics properties and interparticle interactions. CMC-stabilized HFO nanoparticles displayed a much more negative potential surface than starch-stabilized ones did, as CMC is a stronger stabilizer than starch. The ξ potentials for CMC stabilized HFO nanoparticles remained unchanged throughout 15 days of aging.

**S2 synethesis of modified HFO nanoparticles**

100 mL of Fe(NO_3_)_3_•9H_2_O stock solution was added dropwise to a modifier stock solution with continuous stirring to yield a 500 mL mixture containing 100 mg·L^-1^ Fe and 0–0.4 wt % stabilizer. Then, 30 mL of 0.1M KOH stock solution was added to the mixture dropwise to bring the pH to 7–8.

**S3 XRD, SEM, and TEM tests of modified HFO particles**

TEM samples were prepared by applying drops of stabilized nanoparticle suspension to a carbon-coated copper grid and then drying the grid, which was then measured in a JEM-2010 TEM (JEOL Ltd., Tokyo, Japan). For particle size analysis, nanoparticle size in the TEM images was compared with a nanometer scale bar. TEM images of bare HFO particles were obtained by the above procedure, except that the bare HFO particles were sonicated for 10 min before imaging.

The chemical composition and crystallographic structure of the modified–HFO nanoparticles were analyzed by XRD. The solids were freeze-dried under vacuum at −50°C using an FD-81 freeze dryer (Tokyo Rikakikai Co. Ltd., Tokyo, Japan) for 72 h. The XRD unit was equipped with a Cu K-α radiation source (40 kV, 35 mA). The samples were diffracted over a 2θ range from 10° to 90° at a rate of 0.01° / 3 s at room temperature (25°C). XPS measurements were performed with a Kratos Axis Ultra X-ray photoelectron spectrometer (Thermo escalab 250Xi, USA).

DLS tests the dynamic particle size distribution and ζ potential of the nanoparticles in an aqueous solution. Suspensions of HFO nanoparticles prepared with 100 mg·L^-1^ Fe and 0.064 wt % CMC, 0.16 wt% starch, or no modifier were analyzed by measuring ~1 mL of each sample suspension into a special light-scattering cuvette.


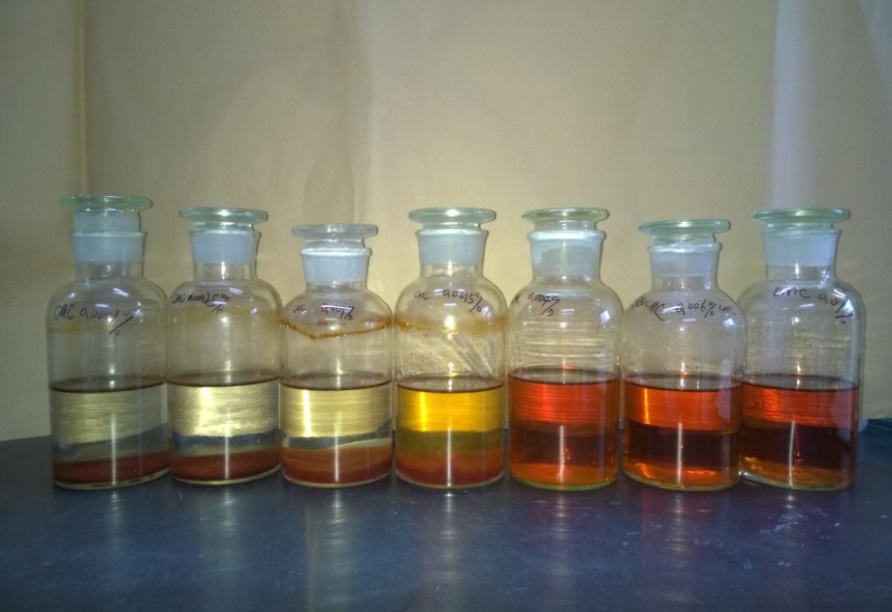


Figure s1 HFO (100 mg·L^-1^ as Fe) nanoparticles prepared in the presence of 0, 0.002, 0.008, 0.016, 0.024, 0.064, and 0.161 wt% CMC (picture taken 15 d after synthesis)


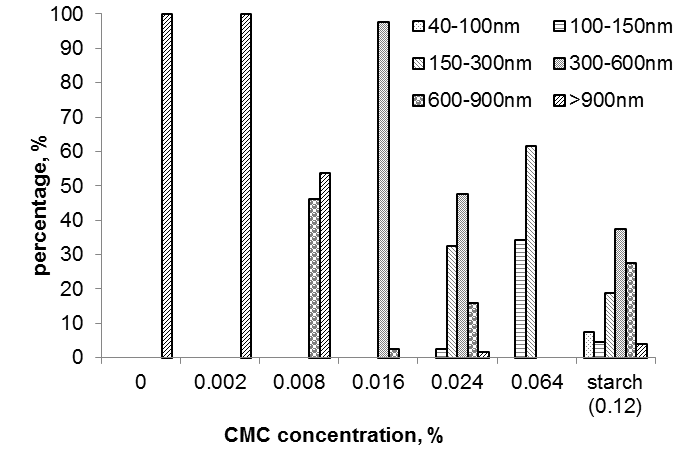


Figure s2 Hydrodynamic size distribution and mean hydrodynamic diameter of HFO nanoparticles prepared at various CMC concentration and 0.12 wt% starch.


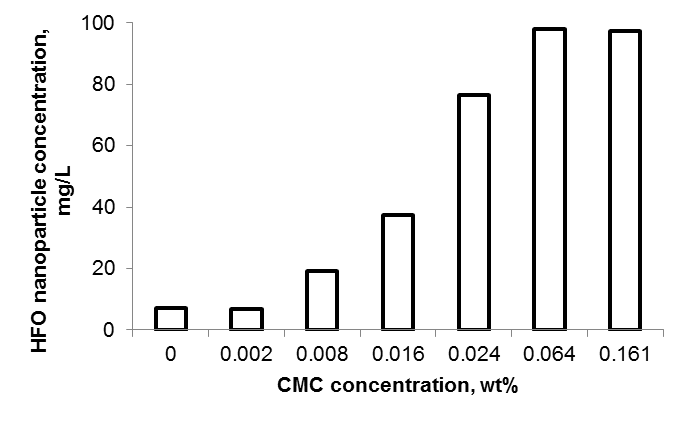


Figure s3 the suspension concentration of HFO nanoparticles at various concentration of CMC after preparation. The samples were taken 15 days after preparation.


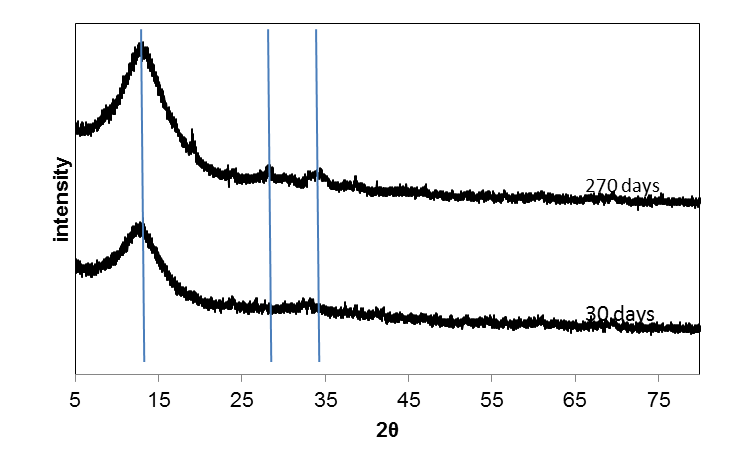


Figure s4 effect of aging time on XRD spectra at pH 7 after 30 d and 270 d. XRD powders were prepared in the conditions: initial As (V) =100 mg·L^-1^, HFO nanoparticles = 300 mg·L^-1^ as Fe.

Figure s5 zeta potential of CMC-HFO as a function of pH.


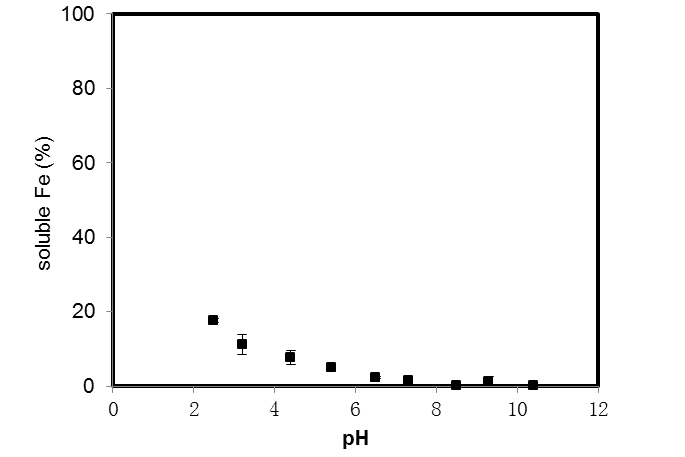


Figure s6 Dissolved Fe concentration as a function of pH. HFO nanoparticles = 100 mg·L^-1^ as Fe.


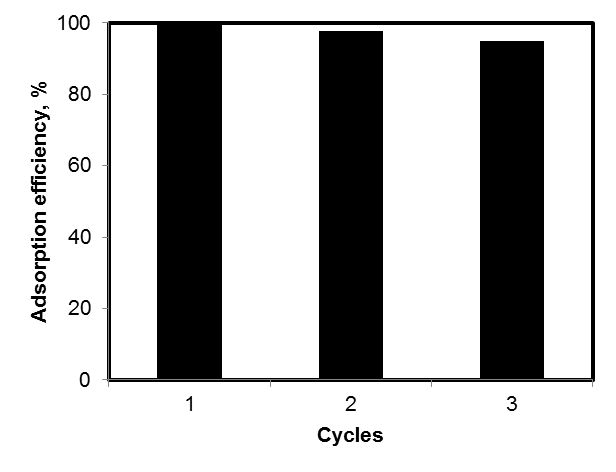


Figure s7 As (V) adsorption efficiency of CMC-HFO regenerated by two regeneration solutions during three successive cycles (HFO nanoparticles = 100 mg·L^-1^ as Fe; initial As concentration = 10 mg·L^-1^; contact time, 72h).


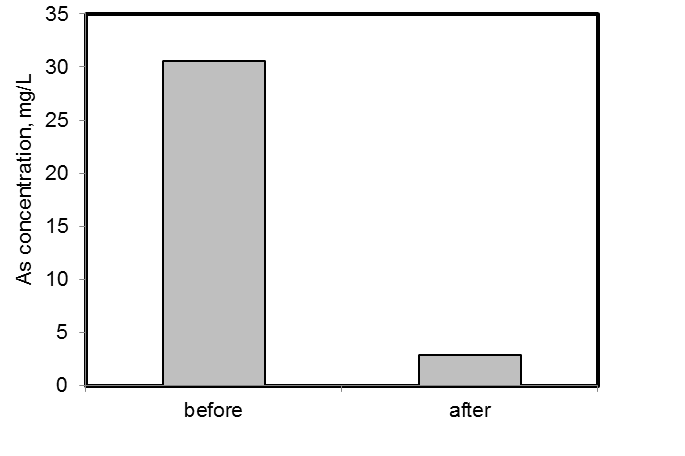


Figure s8. Arsenic uptake of CMC-HFO nanoparticles in real wastewater. The initial arsenic concentration is 38.2 mg·L^-1^. HFO nanoparticles = 100 mg·L^-1^ as Fe.


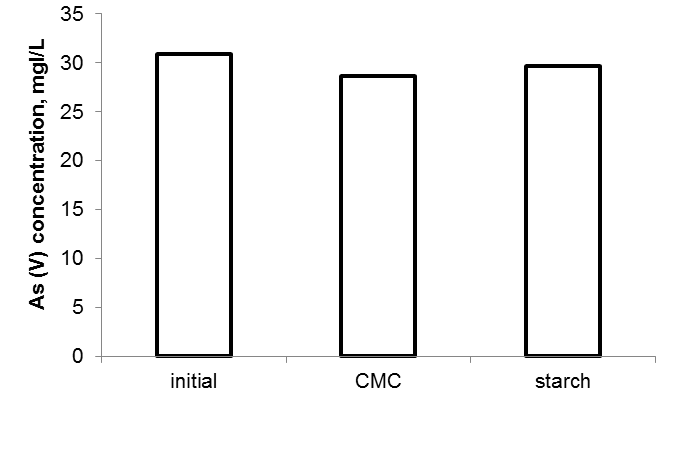


Figure s9 As (V) uptake of CMC and starch (1 wt%). The initial concentration of As (V) is 30.9 mg L^-1^.

Table s1 Composition of real wastewater

|  | Concentration, mg·L^-1^ |
| --- | --- |
| pH | 8.06 |
| Conductivity | 729 µs·cm^-1^ |
| Chloride | 10.7 |
| Sulfate | 103 |
| Nitrate | 2.4 |
| Total As | 38.2 |
| Total P | 12.7 |
| Total N | 7.04 |
| (COD) Chemical oxygen demand | <10 |
| Total Hardness | 167 |
| Total salinity | 565 |
| Dissolved oxygen | 3.2 |
| TSS (Total suspended solids) | 8 |
